# SafAIRway: an airway training for pulmonologists performing a flexible bronchoscopy with nonanesthesiologist administered propofol sedation: A prospective evaluation

**DOI:** 10.1097/MD.0000000000003849

**Published:** 2016-06-10

**Authors:** Melanie Schulze, Bastian Grande, Michaela Kolbe, Sarah Kriech, Christoph B. Nöthiger, Malcolm Kohler, Donat R. Spahn, Daniel Franzen

**Affiliations:** a Institute of Anesthesiology; b Simulation Center, University Hospital Zurich; c ETH Zurich; d Department of Pulmonology, University Hospital Zurich, Zurich, Switzerland.

**Keywords:** airway management, algorithm, bronchoscopy, psychological safety, sedation

## Abstract

Nonanesthesiologist administered propofol (NAAP) sedation for flexible bronchoscopy is controversial, because there is no established airway management (AM) training for pulmonologists. The aim was to investigate the performance and acceptance of a proposed AM algorithm and training for pulmonologists performing NAAP sedation. The algorithm includes using 3 maneuvers including bag mask ventilation (BMV), laryngeal tube (LT), and needle cricothyrotomy (NCT). During training (consisting of 2 sessions with a break of 9 weeks in between), these maneuvers were demonstrated and exercised, followed by 4 consecutive attempts to succeed with each of these devices. The primary outcome was the improvement of completion time needed for a competent airway. Secondary outcomes were the trainees’ overall reactions to the training and algorithm, and the perceptions of psychological safety (PS). The 23 staff members of the Department of Pulmonology performed a total of 552 attempts at AM procedures (4 attempts at each of the 3 maneuvers in 2 sessions), and returned a total of 42 questionnaires (4 questionnaires were not returned). Median completion times of LT and NCT improved significantly between Sessions 1 and 2 (*P* = 0.005 and *P* = 0.04, respectively), whereas BMV was only marginally improved (*P* = 0.05). Trainees perceived training to be useful and expressed satisfaction with this training and the algorithm. The perception of PS increased after training. An AM algorithm and training for pulmonologists leads to improved technical AM skills, and is considered useful by trainees and raised their perception of PS during training. It thus represents a promising program.

## Abstract 2

Supplemental Digital Content is available in the text

## 1 Introduction

Sedation with propofol during flexible bronchoscopy (FB) has been the standard of care for several years, since patient comfort, tolerance, bronchoscopic ease, and willingness to undergo a repeat procedure have been shown in multiple studies.^[1–5]^ Generally, it is considered safe^[6]^ and expert panels recommend moderate sedation with a target Richmond Agitation Sedation Scale ranging between −2 and −3 during FB.^[7–9]^ A growing number of bronchoscopists perform sedation themselves, which has been termed nonanesthesiologist administered propofol (NAAP) sedation. Although NAAP sedation has proved to be feasible, safe, and cost-effective,^[6,10,11]^ there is an ongoing debate regarding NAAP sedation during FB in several countries.^[7,12]^ The most frequent complication during NAAP sedation is respiratory depression and apnea. As a result, according to a recent prospective study, 10% of the patients undergoing a bronchoscopy with NAAP sedation needed unforeseen assistant ventilation due to hypoxia.^[13]^ This emphasizes the need for structured training of airway management (AM), and knowledge of propofol pharmacodynamics.^[1,14]^ Whereas anesthesiologists are familiar with airway algorithms and training,^[15]^ nonanesthesiologists insufficiently focus on these issues during their residency.^[14]^ A recent survey among all Swiss pulmonologists revealed a very low rate of systematic basic education and training in NAAP sedation.^[14]^ Recently it has been shown that airway algorithm training for anesthesiologists, including practicing the use of different airway devices by multiple consecutive attempts with each device, led to significant performance improvement.^[16,17]^ Presently, to our knowledge, there is no study on the development, implementation, and evaluation of systematic AM training for bronchoscopists using NAAP sedation. The purpose of this study was to develop and evaluate a systematic AM training program including an airway algorithm for pulmonologists using NAAP sedation.

## 2 Methods

### 2.1 Subjects

The complete staff of the Department of Pulmonology, consisting of physicians performing FB and bronchoscopy nurses, participated in the training program and the associated study. We recorded age, sex, professional category, and years of experience in anesthesia and intensive care medicine of each of the participants. Approval from the ethics committee was waived, and a declaration of nonobjection was issued by the local ethics committee. All participants gave written informed consent.

### 2.2 AM algorithm

In January 2015, a systematic training program in propofol pharmacodynamics and AM for pulmonologists performing FB was developed and implemented at our institution (SafAIRway). For this purpose, an emergency algorithm for the handling of procedure-related respiratory depression and apnea during FB with NAAP sedation was developed by 4 experienced consultant anesthesiologists and 2 experienced consultant pulmonologists, according to the difficult airway algorithm of the American Society of Anesthesiology,^[15]^ and modified for the special requirements during FB (Fig. 1 A and B). The algorithm is applied when predefined cut-off points are reached, which are fulfilled when either oxygen saturation declines more than 4% from baseline and/or below 90% absolute, or if there is an apnea of more than 30 seconds. According to the study published by Biro et al,^[16]^ the algorithm is linked to 3 airway-saving maneuvers with a semi-sequential approach:

1. bag mask ventilation (BMV) as a basic and noninvasive airway rescue maneuver;
2. laryngeal tube (LT) (VBM, Sulz, Germany); and
3. needle cricothyrotomy (NCT) and ventilation via Cricath and Ventrain (Ventinova Medical, Eindhoven, The Netherlands B.V.).

**Figure 1:**
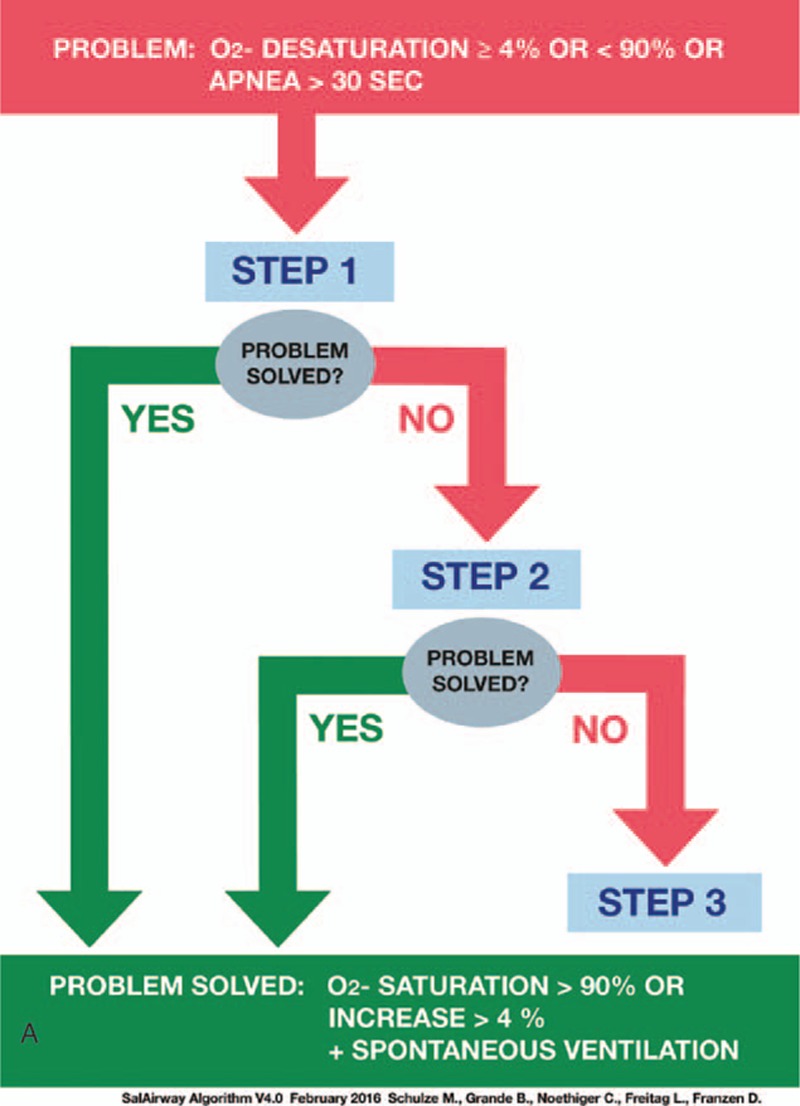
SafAIRway algorithm: SafAIRway algorithm (A) illustrates the semi-sequential approach in case of a relevant oxygen desaturation or apnea during flexible bronchoscopy, which is linked to 3 actions shown in (B). Step 1 covers basic airway management maneuvers not specifically practiced during the course. Step 2 mainly covers bag mask ventilation. In step 3 of the algorithm, after bag mask ventilation has failed to succeed, laryngeal tube placement, cricothyrotomy, or bronchoscopic intubation are proposed.

**Figure 1 (Continued):**
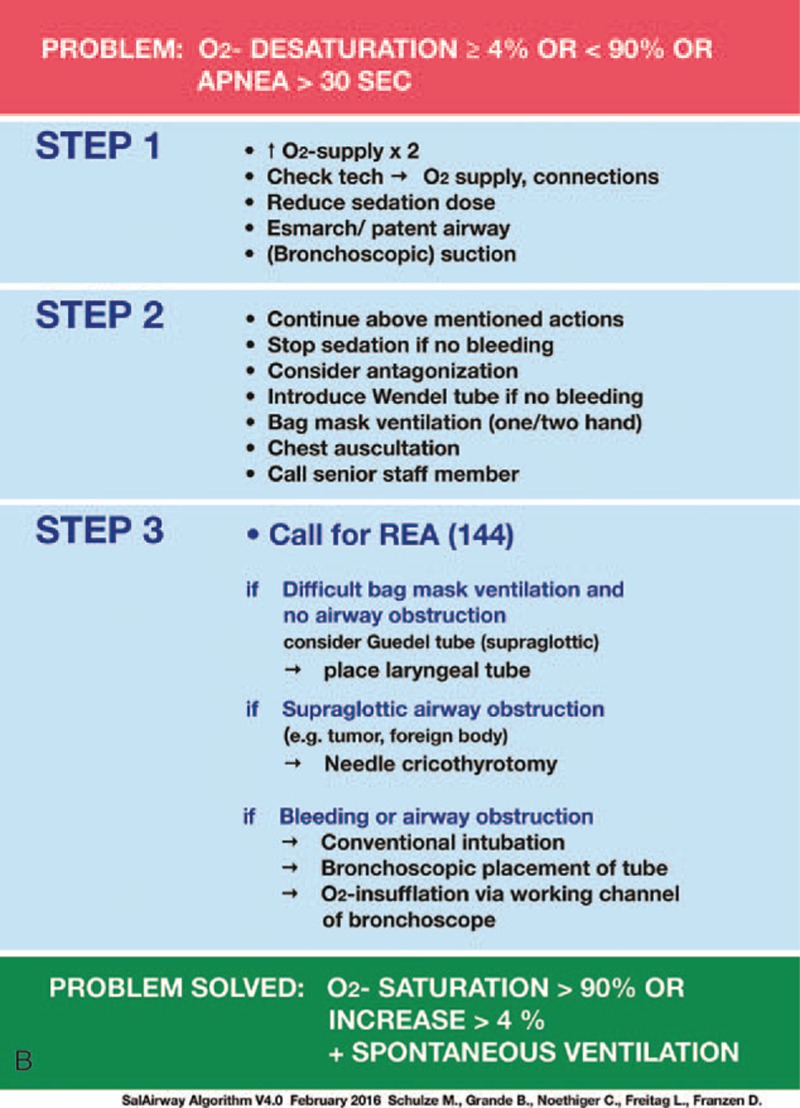
SafAIRway algorithm: SafAIRway algorithm (A) illustrates the semi-sequential approach in case of a relevant oxygen desaturation or apnea during flexible bronchoscopy, which is linked to 3 actions shown in (B). Step 1 covers basic airway management maneuvers not specifically practiced during the course. Step 2 mainly covers bag mask ventilation. In step 3 of the algorithm, after bag mask ventilation has failed to succeed, laryngeal tube placement, cricothyrotomy, or bronchoscopic intubation are proposed.

The 1st of 3 technical steps in this algorithm is BMV. If BMV fails, an LT is inserted as the 2nd technical step. As a potential 3rd technical step, or if the patient is found to have a supraglottic airway obstruction, an NCT will be performed.

### 2.3 AM training program

SafAIRway was scheduled for 2 training days (Sessions 1 and 2) with a break of approximately 9 weeks in-between. The courses were held by 1 of 2 consultant anesthesiologists (both members of the “Difficult Intubation Drill” instructor team at our institution) using a standardized protocol. All courses were observed by a senior pulmonologist who did not participate in the course. An intubation manikin (Laerdal Airway Management trainer, Stavanger, Norway) was positioned in a standard “sniffing” position. During the 1st session, the participants received an oral presentation on the algorithm, basic airway knowledge, details of the airway devices used in the algorithm, and pharmacodynamics of propofol. The instructor demonstrated each of the 3 airway-saving maneuvers step by step. Thereafter, the participants had the opportunity to practice each technique once on the manikin. This was followed by 4 consecutive attempts to perform each technique, for which success and completion times (times needed to succeed in establishing a competent airway) as a measure of clinical performance were recorded for each attempt. Median times of all participants for each of the 4 attempts were used for the analysis. The 2nd session, which was scheduled 9 weeks after Session 1, began with a short introduction to re-familiarize participants with the devices. Shortly thereafter, 4 consecutive attempts applying the 3 AM interventions were again undertaken. Only successful attempts were included in the comparison between the 2 sessions. In the following report, the 1st training day will be called Session 1, and the 2nd training day will be called Session 2. Likewise, the 1st and the 4th attempt of each session will be referred to as Attempts 1 and 4, respectively.

### 2.4 Study design and outcomes

This study was conducted as a prospective longitudinal study. The primary outcome was the success rate and improvement of completion time to establish a competent airway with the respective procedure between the 1st and the 2nd sessions, and between the 1st and 4rth attempts of each session. Success was defined as a correctly placed device with a consecutive thoracic movement of the manikin upon ventilation within 1 min. Completion time was defined as the time in seconds needed to establish the described success. For each attempt, picking up the advice was defined as the start time. The finish time was defined as the time when a 1st thoracic movement was observed by the instructor. Attempts taking longer than 1 min were counted as failure. Secondary outcomes were trainees’ reactions to the training and algorithm and their perceptions of psychological safety (PS) during the training. For this, the participants were asked to complete a questionnaire to measure operator comfort before and after training of both sessions (see Appendix). All questionnaires were handled anonymously. To assess trainees’ overall reactions to the training, we used a German version of a scale measuring trainee evaluation of the training.^[18]^ This scale contained 9 items, which were rated on a 6-point Likert scale ranging from 1 (strongly disagree) to 6 (strongly agree) and were presented to the trainees after the course. Reliability analyses of trainee agreement resulted in excellent Cronbach α values of 0.91. Seven open questions, such as “What did you particularly like?” were also provided (Table 1). The algorithm was rated by means of a postquestionnaire using a 6-point scale ranging from 1 (strongly disagree) to 6 (strongly agree) in 3 items, according to a recent study using similar questions concerning the assessment of a safe surgery checklist.^[19]^ To assess PS during the training, the validated German translation of Edmondson PS scale^[20]^—adapted to the training context—was applied before and after the course. Items were rated on a 5-point Likert scale ranging from 1 (strongly disagree) to 5 (strongly agree). Reliability analysis results in acceptable Cronbach values of 0.73 and 0.79, respectively.

**Table 1:**
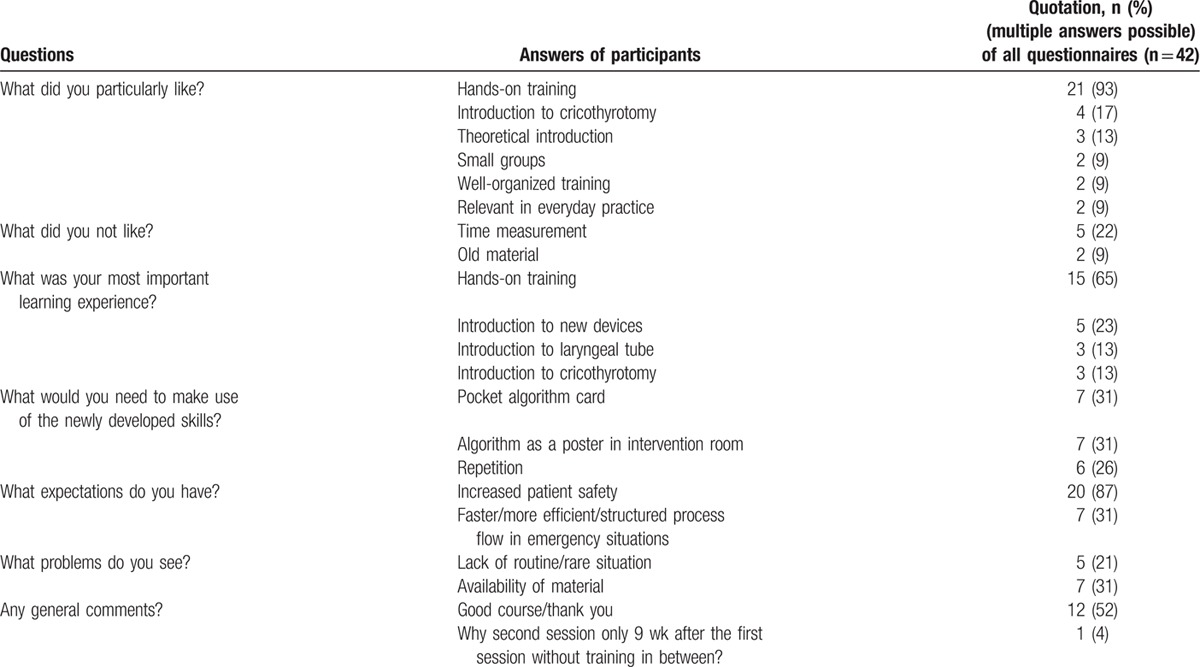
Open questions on perceptions of the training with participants’ answers.

### 2.5 Statistical analysis

Continuous variables are displayed as median (interquartile range [IQR]), and categorical variables as absolute values (percentages). Correlation of covariates such as age, professional category, and years of experience to completion times was assessed using the Kendall tau test. Changes in completion times between Sessions 1 and 2, and between the 1st and 4th attempts, and reactions to the training and algorithm, and levels of PS were tested using the Wilcoxon matched-pair, signed-rank test. A 2-sided *P* value <0.05 was considered statistically significant for all outcomes. All analyses were performed using IBM SPSS Statistics for Windows, version 22.0 (IBM Corporation, Armonk, NY).

## 3 Results

Twenty-three staff members of the Department of Pulmonology (18 physicians and 5 nurses) performed a total of 552 standardized airway rescue procedures between March 2015 and August 2015, and returned a total of 42 questionnaires (4 questionnaires were not returned). Of all participants, 15 (64%) were male. The median age was 39 (IQR 35, 44: range 19–61). Among the physicians, 10 (43%) were senior staff members and 8 (35%) were residents. Median pre-existing practical experience of the participants in anesthesiology and intensive care medicine was 0 (IQR 0, 0; range 0–9) and 6 (IQR 3, 12; range 0–96) months, respectively. Previous experience in BMV, LT placement, and cricothyrotomy was reported by 96%, 9%, and 4%, respectively. Session 2 was conducted after a median of 9 weeks after Session 1.

### 3.1 Primary outcomes

**Figure 2:**
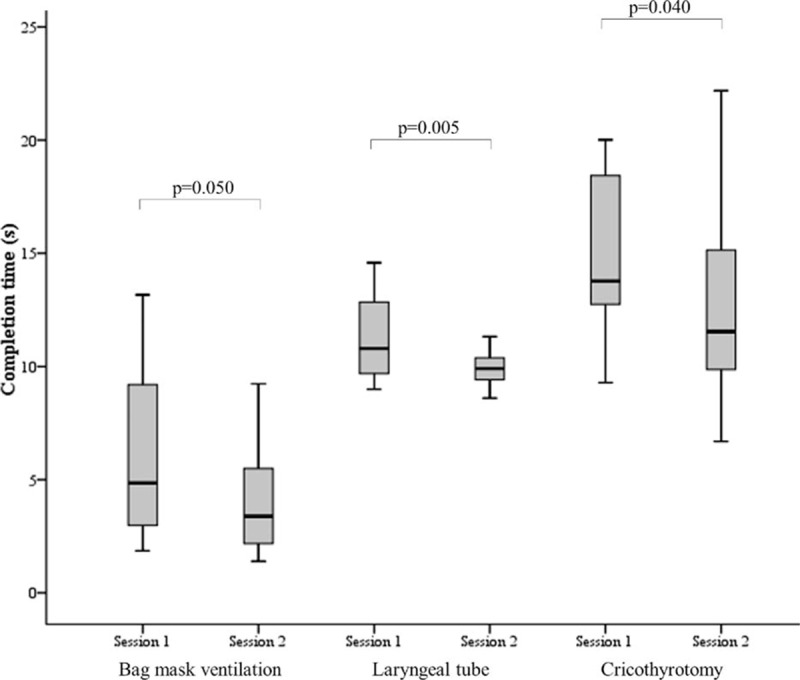
Median completion times in training Sessions 1 and 2. Boxplots of median completion times of all 3 airway management maneuvers in Session 1 compared with Session 2. Dark horizontal lines represent the median, with the box representing the 25th and 75th percentiles, the whiskers the 5th and 95th percentiles. *P* values are estimated using the Wilcoxon matched-pair, signed-rank test.

The success rate for all performed procedures was 100%. Median completion times of all procedures on the 1st compared with 4th attempt of both training sessions are shown in Table 2. All completion times did not significantly correlate to sex, age, professional category, and pre-existing practical experience of the participants in anesthesiology or intensive care medicine. At both sessions, the completion times of all airway rescue skills improved significantly between the 1st and 4th attempts (Table 2). In Session 1, the median (IQR) completion times of all 4 attempts for BMV, LT placement, and cricothyrotomy was 4.9 (3.0, 9.1), 10.8 (9.7, 12.7), and 14.5 (12.8, 18.4) s, respectively, compared to Session 2 with 3.6 (2.2, 6.3), 9.9 (9.4, 40.4), 11.3 (9.9, 15.1), s, respectively. The improvement of the median completion times of LT placement and cricothyrotomy was significant between Sessions 1 and 2 (*P* = 0.005 and *P* = 0.04, respectively), whereas improvement of completion time of BMV was only marginally significant (*P* = 0.05) (Fig. 2).

**Table 2:**
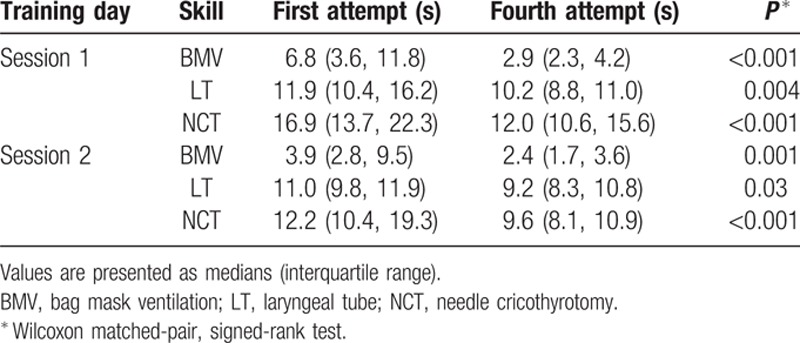
Completion times of airway rescue maneuvers on the 1st compared with the 4rth attempt and median completion time of all attempts of both training sessions.

### 3.2 Secondary outcomes

**Figure 3:**
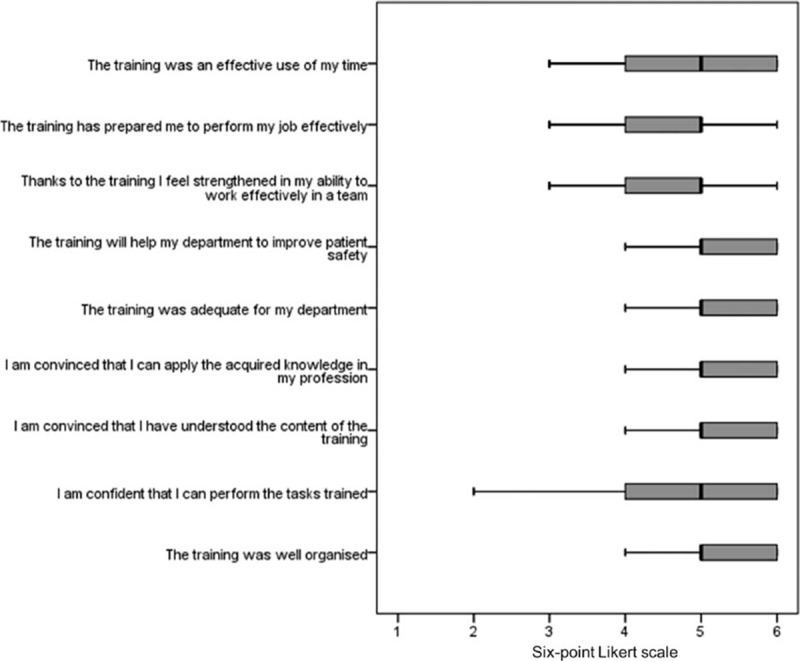
Trainees’ overall reactions to the training. Boxplots of median overall reactions by the trainees to the training. The Y-axis represents the 9 items, which were rated on a 6-point Likert scale ranging from 1 (strongly disagree) to 6 (strongly agree) displayed on the X-axis. Dark vertical lines represent the median, with the box representing the 25th and 75th percentiles, the whiskers the 5th and 95th percentiles.

Considering the 42 questionnaires returned by the 23 participants, the course was evaluated positively with a median rating of 5.1 (4.8, 5.6) points on the 6-point Likert scale (Fig. 3). However, we found a significant correlation between sex and reactions to the training. Females expressed significantly (r_τ_ = 0.30, *P* = 0.02) more positive reactions (5.6; IQR 5.0, 6.0) to the training than male participants (5.0; IQR 4.6, 5.3). Other covariates such as age, professional category, and pre-existing experience in anesthesiology or intensive care medicine did not significantly correlate to reactions to the training (all r_τ_ < 0.054, *P* > 0.05). Answers of the participants to open questions with multiple possible answers are summarized in Table 1. The newly developed AM algorithm was also evaluated positively with a median rating of 5.0 (4.7, 6.0) points on the 6-point Likert scale. Overall, participants reported high levels of PS during all training days (median rating 4.4; IQR 3.9, 4.6 points on the 5-point Likert scale). Notably, there were significantly higher levels of PS after training (median rating 4.4; IQR 4.0, 4.9) compared with before (median rating 3.7; IQR 3.4, 3.8) (*P* = 0.02).

## 4 Discussion

There is an ongoing debate on the optimal mode of sedation during FB. Yet NAAP sedation during FB remains controversial.^[7,12]^ A major concern of NAAP sedation is insufficient education in AM, since the most frequent complication during NAAP sedation is respiratory depression and apnea in up to 10% of patients undergoing FB.^[13]^ A recent survey among Swiss pulmonologist revealed a lack of such training.^[14]^ Thus investigation toward safe application of NAAP sedation for FB is crucial.

In the present study, we aimed to implement and investigate a novel AM algorithm for pulmonologists performing NAAP sedation, set up within a systematic airway training course (SafAIRway), and tested its efficacy in terms of technical abilities, satisfaction, and perceptions of PS.

According to others,^[21]^ clinical performance of the participants was assessed using completion times to succeed with each of the 3 AM maneuvers. We found significant learning curves with markedly improved completion times for each device within the 4 attempts of both sessions. Similar results have been recently shown in an airway training study, but with attendance of anesthesiologists only.^[16]^ In the SafAIRway program, the focus was BMV, LT placement, and cricothyrotomy using Cricath and Ventrain. Certainly, alternative AM (e.g., laryngeal mask) devices could have been used for this program. However, the LT has been assessed as the most successful and safe supraglottic airway device for nonexperts,^[22–24]^ and the usefulness of this device in securing a difficult airway has been shown in various studies, even in cases where insertion of the laryngeal mask had failed.^[25,26]^ Using Ventrain, minute ventilation and avoidance of high airway pressures were superior compared with traditional hand-triggered jet ventilation systems, particularly when complete upper airway obstruction occurs.^[27]^

The SafAIRway program consisted of 2 AM training sessions, separated by a median of 9 weeks in between. We aimed to investigate whether a repeated session after a break of several weeks might have an impact on trainee performance. We observed a significant improvement in completion times for LT placement and cricothyrotomy between both sessions. For BMV, the improvement was only marginally significant. Presumably this is due to the complexity of LT placement and cricothyrotomy with the least pre-existing experience, which might have led to heightened focus from such participants. In contrast, a well-known and already well-practiced procedure like BMV might not have been possible to improve as dramatically in repeated sessions. Notably, there is uncertainty about the impact that simulation or training may have on a trainee learning the desired knowledge, skills, attitudes, and behavior, and about how much of what is learned during training is actually manifest during patient care.^[28]^ However, the results underline the need for repeated training. For future studies, it will be interesting to investigate whether a shorter or longer break between sessions has an impact on performance improvement.

Participants demonstrated high levels of PS during the training, which is known to have a positive effect on learning.^[29,30]^ Moreover, those levels of PS even increased after training. PS describes perceptions of the consequences of taking interpersonal risks in a particular context such as the workplace; for example, whether safety-related doubts can be expressed without fear of being judged. Studies have shown that this parameter is a critical measure for learning behavior.^[29,30]^ The SafAIRway algorithm has been well received, and feedback about this course via the above-mentioned questionnaires was overwhelmingly positive, with an expressed demand for repeated courses. Along with the reported high levels of PS during training and increased levels of PS after training, these results are in accordance with our expectation that the established training course may be a valuable learning instrument. Furthermore, a systematic AM algorithm and training like SafAIRway may be a valuable tool to counter controversies regarding NAAP sedation even though the proposed algorithm and the associated airway training were specifically developed for pulmonologists performing NAAP sedation. However, the conclusions might be transferred to other specialties performing NAAP sedation (i.e., gastroenterologists).

This study has some limitations. First, it was conducted at a single center, and performed on a small case number. Second, since the airway algorithm was developed as a consensus of local experts of different specialties, it does not represent general evidence and may not be transferable to other countries or hospitals.

## 5 Conclusion

This airway training course for pulmonologists, including a novel airway algorithm for bronchoscopies under NAAP sedation, leads to improved technical airway skills, widespread satisfaction, and increased levels of PS among participants. It is thus a promising program and may be a valuable tool for ensuring patient safety during NAAP sedation.

## Acknowledgments

We thank Prof. Lutz Freitag (Department of Pulmonology, University Hospital Zurich, Switzerland) for his critical advice on the implantation of SafAIRway and statistical guidelines, and Mrs Irene Odermatt (Institute of Anesthesiology, University Hospital Zurich, Switzerland) for helping us with the layout of the SafAIRway algorithm.

## Supplementary Material

Supplemental Digital Content
