# Supplementary material for: SafAIRway: an airway training for pulmonologists performing a flexible bronchoscopy with nonanesthesiologist administered propofol sedation: A prospective evaluation
Source: Medicine (Baltimore). 2016 Jun 10;95(23):e3849. doi: 10.1097/MD.0000000000003849 (PMC4907671; doi:10.1097/MD.0000000000003849)
Supplement: Supplemental Digital Content [file medi-95-e3849-s001.docx]

**Appendix**

Psychological safety

|  | strongly disagree | disagree | undecided | agree | strongly agree |
| --- | --- | --- | --- | --- | --- |
| If you make a mistake on this team, it is often held against you |  |  |  |  |  |
| Members of this team are able to bring up problems and tough issues |  |  |  |  |  |
| People on this team sometimes reject others for being different |  |  |  |  |  |
| It is safe to take a risk on this team |  |  |  |  |  |
| It is difficult to ask other members of this team for help |  |  |  |  |  |
| No one on this team would deliberately act in a way that undermines my efforts |  |  |  |  |  |
| Working with members of this team, my unique skills and talents are valued and utilised |  |  |  |  |  |

Algorithm

How do you evaluate?

|  | very bad | bad | rather bad | rater good | good | very good |
| --- | --- | --- | --- | --- | --- | --- |
| ... the purpose of the difficult airway algorithm to describe ? |  |  |  |  |  |  |
| … the difficult airway algorithm to use ? |  |  |  |  |  |  |
| … attaching any suggestions how to use the difficult airway algorithm ? |  |  |  |  |  |  |

Evaluation

|  | strongly disagree | disagree | slightly disagree | slightly agree | agree | strongly agree |
| --- | --- | --- | --- | --- | --- | --- |
| The training was well organised |  |  |  |  |  |  |
| I am confident that I can perform the tasks trained |  |  |  |  |  |  |
| I am convinced that I have understood the content of the training |  |  |  |  |  |  |
| I am convinced that I can apply the acquired knowledge in my profession |  |  |  |  |  |  |
| The training was adequate for my department |  |  |  |  |  |  |
| The training will help my department to improve patient safety |  |  |  |  |  |  |
| Thanks to the training I feel strengthened in my ability to work effectively in a team |  |  |  |  |  |  |
| The training has prepared me to perform my job effectively |  |  |  |  |  |  |
| The training was an effective use of my time |  |  |  |  |  |  |

Open questions:

1. What did you particularly like?
2. What did you not like?
3. What is the most important learning experience for you?
4. What do you need that you can use the learned skills in your daily work?
5. General comments and notes:
